# Autoimmune causes of encephalitis syndrome in Thailand: prospective study of 103 patients

**DOI:** 10.1186/1471-2377-13-150

**Published:** 2013-10-20

**Authors:** Abhinbhen Saraya, Aekkapol Mahavihakanont, Shanop Shuangshoti, Nuntaporn Sittidetboripat, Tayard Deesudchit, Michael Callahan, Supaporn Wacharapluesadee, Henry Wilde, Thiravat Hemachudha

**Affiliations:** 1 Neuroscience Centre for Research and Development, Division of Neurology, Department of Medicine, Faculty of Medicine, Chulalongkorn University, Bangkok, Thailand; 2 King Chulalongkorn Memorial Hospital, Bangkok, Thailand; 3 Neuroscience Centre for Research and Development, Department of Pathology, Faculty of Medicine, Chulalongkorn University, Bangkok, Thailand; 4 Department of Pediatrics, Faculty of Medicine, Chulalongkorn University, Bangkok, Thailand; 5 Division of Infectious Diseases, Massachusetts General Hospital, Boston, MA, USA; 6 Harvard Medical School, Boston, MA, USA

**Keywords:** Encephalitis, Autoimmune encephalitis, Paraneoplastic encephalitis, Limbic encephalitis

## Abstract

**Background:**

Data on encephalitis in Thailand have not been completely described. Etiologies remain largely unknown. We prospectively analyzed 103 Thai patients from 27 provinces for the causes of encephalitis using clinical, microbiological and neuroimaging indices; caseswithout a diagnosis were evaluated for autoimmune causes of encephalitis.

**Methods:**

Patients with encephalitis and/or myelitis were prospectively studied between October 2010 and August 2012. Cases associated with bacterial, rickettsial and mycobacterial diseases were excluded. Herpes viruses 1-6 and enteroviruses infection was diagnosed using PCR evaluation of CSF; dengue and JE viruses infection, by serology. The serum of test-negative patients was evaluated for the presence of autoantibodies.

**Results:**

103 patients were recruited. Fifty-three patients (52%) had no etiologies identified. Twenty-five patients (24%) were associated with infections. Immune encephalitis was found in 25 (24%); neuropsychiatric lupus erythematosus (4), demyelinating diseases (3), Behcet’s disease (1) and the remaining had antibodies to NMDAR (5), ANNA-2 (6), Yo (2), AMPA (1), GABA (1), VGKC (1) and NMDA coexisting with ANNA-2 (1). Presenting symptoms in the autoimmune group included behavioral changes in 6/25 (versus 12/25 in infectious and 13/53 in unknown group) and as psychosis in 6/25 (versus 0/25 infectious and 2/53 unknown). Seizures were found in 6/25 autoimmune, 4/25 infectious and 19/53 unknown group. Two patients with anti-ANNA-2 and one anti-Yo had temporal lobe involvement by magnetic resonance imaging. Two immune encephalitis patients with antibodies to NMDAR and ANNA-2 had ovarian tumors.

**Conclusions:**

Autoantibody-associated encephalitis should be considered in the differential diagnosis and management algorithm regardless of clinical and neuroimaging features.

## Background

The priority in managing encephalitis is to first exclude infectious causes, many of which are effectively treated using antimicrobial agents. Non-infectious causes of encephalitis due to autoimmune and paraneoplastic etiologies, are also a diagnostic priority as these syndromes may be life-threatening, are often associated with an underlying malignancy or systemic disease, and may be treatable if diagnosed early. Despite dramatic advances in identifying the protean causes of encephalitis, a significant proportion of cases still defy diagnostic work-up. Recent international studies reported greater than 50% of encephalitis patients as have no etiology identified, despite extensive evaluation [1,2]. The California Encephalitis Project (CEP) initiated in 1998 [3] reported that 25% of 334 patients had confirmed or possible infectious etiologies, whereas 208 cases (62%) lacked a diagnosis. Although patients with lymphocytic and normal glucose cerebrospinal fluid (CSF) profile are usually suspected to have a viral meningoencephalitis, there is growing evidence of another entity capable of producing an aseptic CSF profile autoantibody-associated encephalitis.

In 2004, Thieben et al published a series of 7 patients with potentially reversible autoimmune limbic encephalitis associated with neuronal potassium channel antibody [4]. Vincent et al reported another 10 cases with limbic encephalitis associated with low plasma sodium that were identified as potassium channel antibody-associated encephalopathy [5]. These publications are among the earliest studies to emphasize the significance of neuronal autoantibodies in aseptic encephalitis. After the discovery of antibody to N-methyl-D-aspartate receptor (NMDAR) in 2007 [6], there have been additional reports of immune-mediated encephalitis. The first case series of anti-NMDAR limbic encephalitis included 12 women with prominent psychiatric symptoms [6]. Ten of 20 encephalitis patients with unidentified etiologies in CEP (The California Encephalitis Project) were later found to have anti-NMDAR antibody [7]. These patients (6 females and 4 males) were young adults (mean age 18.5 year) who primarily presented with dyskinesia and psychiatric manifestations [7]. Subsequent CEP study (2007-2010) demonstrated that encephalitis associated with anti-NMDAR antibody (32 of 761) was nearly as common as viral causes (47 of 761) in patients less than 30 years of age [8]. Seizures, language and autonomic dysfunctions, movement disorder and psychoses were predominating clinical features in these patients [9].

A report from Malaysia [10] revealed that 8 of 16 adult encephalitis patients (14-29 years of age) and 2 children (both 9 years old) with dominant psychiatric features had anti-NMDAR antibody yet lacked evidence of underlying tumor. Autoantibody-associated encephalitis other than anti-NMDAR is now increasingly reported [7,8]. These patients can present with a wide range of manifestations unrelated to solid tumors and are distributed across a wider age group. These reports have confirmed the incidence and severity of immune-mediated encephalitis in non-infectious cases of encephalitis.

We prospectively studied 103 Thai patients between 2 and 85 years of age, presenting with clinically non-bacterial, non-rickettsial, non-TB, non-fungal and non-parasitic encephalitis and/or myelitis with normal or lymphocytic CSF profile who were evaluated by the Neurology service of King Chulalongkorn Memorial Hospital (KCMH) between October 2010 and August 2012. Eleven pediatric patients aged one to fourteen years seen over the same period were included in this study.

## Methods

### Study design

This prospective study of patients with clinical evidence of encephalitis was conducted at KCMH, a tertiary referral hospital. Seventeen hospitals in Bangkok, Chonburi, Nakhonpatom and Ayutthaya provinces recruited patients. The study was approved by the KCMH ethics committee (reference number 015/2011). Informed consent was obtained from all patients in written form; when patients were impaired or underage, consent was obtained from a family member, parent or guardian. Only patients with clinical evidence of encephalitis and/or myelitis with or without peripheral nerve involvement were included. Patients with laboratory evidence of infectious encephalitis, e.g. viral, bacteria, mycobacterium tuberculosis (TB), parasite or rickettsia were excluded. Only patients who were normocellular or showed lymphocytosis with normal or slightly decreased glucose in CSF were enrolled. Patients with low CSF glucose and who had no evidence of viral, bacterial, TB, fungal, or parasitic infection and patients without evidence of malignant cells after cytocentrifugation were included. Magnetic resonance imaging (MRI) of the brain and/or spinal cord was performed within 48-72 hours of admission. In patients with unstable clinical conditions, computed tomography (CT) scan was performed instead of MRI.

### Definition

Encephalitis was defined as a clinical syndrome comprising of headache, impaired cognitive function or consciousness, seizures or other focal deficits in the presence of an imaging study consistent with brain dysfunction. Myelitis was also included since it may be a sole manifestation or presenting feature subsequently followed by involvement of other structures. Myelitis was defined as fever and/or evidence of spinal cord dysfunction manifesting as sensori-motor deficits or pure motor deficit with or without sphincter involvement. Onset of the disease was defined as acute when neurological symptoms appeared at onset of any symptoms or within 7 days of a prodrome. They were subacute when appearing 8-30 days after prodrome and chronic if the interval was longer than 30 days.

All suspected cases of central nervous system infection with bacteria, fungi, parasites or mycobacteria were excluded as well as encephalopathy secondary to sepsis or systemic inflammatory response syndrome.

### Investigations

Investigations included routine CBC, BUN, Cr, serum electrolytes, HIV antibody, chest X-Ray and liver function studies. Blood cultures were performed in all febrile case. Immunofluorescence assays for rickettsia were performed on serum when appropriate. All CSF was examined using cytology, glucose and protein levels, and cryptococcal antigen determination. PCR was used to evaluate CSF for herpes simplex virus (HSV) varicella zoster virus (VZV), Epstein Barr virus (EBV), cytomegalovirus (CMV), human herpes virus 6 (HHV-6) and enteroviruses. Patients with a history of animal bites and/or those with signs or symptoms suggesting rabies, had saliva, urine, CSF and extracted hair follicles tested for rabies viral RNA. Serologies for Japanese Encephalitis virus (JEV) and dengue virus were performed using IgM capture methods [11]. When indicated by MRI and by initial-negative serology studies, patients were evaluated for Nipah, Hendra and West Nile Virus (WNV) by PCR and paired acute and convalescent serology studies. MRI was performed for all cases unless the patient was unstable. MR studies were performed with a 3-T MR imager (Phillip MR systems Achieva Release 2.6.3.7) at Phyathai 2 Hospital and a 3-T Horizon MR imager (GE Medical systems) at KCMH. Contrast-enhanced studies were obtained using intravenous gadopentetate dimeglumine.

### Detection of autoantibodies

Examination for autoantibodies was performed on archived serum of all patients admitted between October 2010 and June 2011 who presented with a negative work-up for infectious etiologies. Routine autoantibody testing was initiated in June 2011 after infection had been ruled out by laboratory studies and extended culture. Testing for autoantibodies was completed within 7-14 days. Twenty autoimmune and paraneoplastic neurological syndrome (PNS) related antibodies consisting of anti-NMDAR, anti-AMPA (2-amino-3-(5-methyl-3-oxo-1,2- oxazol-4-yl) propanoic acid)-1 and 2 receptor, anti-CASPR2 (contactin-associated protein 2), anti-LGI-1(Leucine-rich, glioma inactivated 1), anti-GABA (gamma-aminobutyric acid)-A and B receptor, anti-Hu (ANNA-1), anti-Ri (ANNA-2), anti-Yo (PCA-1), PCA-2, anti-Tr, anti-MAG, anti-myelin, anti-GAD, anti-CV2 (CRMP5), anti-ampiphysin, anti-neuroendothelium, anti-GFAP, anti-synaptophysin and AGNA/anti-SOX1 were determined by indirect immunofluorescence (IIF) assay in sera using EUROIMMUN® (Germany). Briefly, serum was diluted 10-fold and incubated on slides containing either individual antigens expressed in HEK cells or specific tissues (cerebellum, pancreas, intestine and nerve cell) followed by the second antibody conjugated with fluorescence isothiocyanate (FITC). Immunofluorescence was assessed using an inverted fluorescence microscope (Olympus®, model IX81). Assay for neuromyelitis optica (NMO) antibody was performed using standard IIF technique [12]. The diagnosis was confirmed using immunoblot analysis against five autoantibodies (antibodies against anti-amphiphysin, anti CV2, anti-Ri, anti-Yo and anti-Hu). Patient’s serum was diluted 1:100 and incubated with coated test strips followed by co-incubation of enzyme conjugate with alkaline phosphatase labeled anti-human IgG and substrate. The incubated test strips were evaluated using the EUROLineScan® software provided by the manufacturer.

## Results

One-hundred eleven patients with encephalitis and/or myelitis were enrolled between October 2010 and August 2012. Sixty-six patients were admitted to KCMH and 45 represented referrals from 17 hospitals, primarily central Thailand. Eight patients were excluded after investigation showed alternative diagnoses (cerebral infarction, metabolic encephalopathy, cerebral venous thrombosis and subarachnoid hemorrhage). Of 103 patients, presentations included encephalitis (82,79.6%), myelitis (11,10.7%), encephalomyelitis (4,3.9%), meningoencephalitis (3,2.9%), encephalomyeloradiculitis (2, 1.9%) and myeloradiculitis (1,1%). Demographic data are summarized in Table 1.

**Table 1:** Demographic data

**Demographic data**	**Unknown cause (53)**	**Immune-mediated cause (25)**	**Infectious cause (25)**
Category: meningoencephalitis	1(2%)	0	2(8%)
: encephalitis	41(77%)	20(80%)	21(84%)
: myelitis	7(13%)	3(12%)	1(4%)
: encephalomyelitis	3(6%)	0	1(4%)
: encephalomyeloradiculitis	1(2%)	1(4%)	0
: myeloradiculitis	0	1(4%)	0
Age range: 0–15 yr	4(8%)	4(16%)	3(12%)
: 16–60 yr	40(76%)	15(60%)	17(68%)
: 61–90 yr	9(16%)	6(24%)	5(20%)
Male: female	32: 21	8: 17	12: 13
Underlying diseases			
• Normal	38(72%)	15(60%)	19(76%)
• Malignancy	3(6%)	1(4%)	0
• HIV seropositive	2(4%)	0	4(16%)
• Autoimmune disease	1(2%)	5(20%)	1(4%)
• Diabetes mellitus	3(6%)	2(8%)	1(4%)
• Hematologic disease	0	1(4%)	0

Patients were categorized into infectious, immune-mediated and unidentified groups. There were 25 patients (24.3% of 103) in the infectious group; HSV-1 (6,24%), VZV (4,16%), JEV (3,12%), fungi (2,8%), mycobacterium tuberculosis (2,8%), EBV (2,8%), Clostridium tetani (2,8%), bacteria (1,4%), dengue (1,4%), rabies (1,4%), and dual infections with HSV-1 and dengue virus (1,4%). Four cases of TB (2) and fungi (2) were included in this study because they all had atypical presentations that we could not distinguish initially from other viral encephalitis cases. As for TB cases, one presented with acute onset of fever, headache and prominent behavioral changes resembling viral encephalitis. The other patient with TB presented with acute fever, drowsiness accompanied by hemiparesis shortly after onset which was not common in TB cases. None had abnormal chest X-Rays or meningeal enhancement particularly at basal cisterns on CT scan of the brain. The CSF white blood cell counts were 1,050 and 162 cells/mm^3^ respectively with predominant mononuclear cells and both had CSF protein <200 mg/dl with a sugar level of 12 and 40 mg/dl respectively. PCR for TB in CSF was positive in both cases. Two patients with fungal infections also had atypical manifestations. They presented with acute alterations of behavior in one case and brainstem encephalitis with cranial nerve palsies in another. CSF findings showed white blood cells of 9 and 327 cells/mm^3^, sugar levels of 52 and 33 mg/dl, protein levels of 106 and 61 mg/dl respectively. CSF India Ink preparations were negative.

The immune-mediated group comprised of 25 cases (24.3% of 103) consisting of anti-Ri (ANNA2) (6,24%), anti-NMDAR (5,20%), neuropsychiatric lupus erythematosus (NPLE) (4,16%), demyelinating disease (3,12%), anti-Yo (PCA-1) (2,8%), neuro-Behcet’s disease (1,4%), anti-AMPAR (1,4%), anti-GABAR (1,4%), anti-VGKC (1,4%), and co-existence of anti-NMDAR with anti-Ri (1,4%). Three demyelinating cases were newly diagnosed with first attacks of multiple sclerosis or neuromyelitis optica. All presented with encephalitis syndrome. We could not differentiate them clinically from other viral or autoimmune causes.

Comparison of clinical course, neuroimaging studies and results of the workup for immune-mediated and unidentified etiologies are summarized in Table 2.

**Table 2:** Clinical course & investigations

**Clinical course & investigations**	**Unknown cause**	**Immune-mediated cause**	**Infectious cause**
Prodrome symptoms			
• Not clearly defined	19(36%)	8(32%)	2(8%)
• Headache	1(2%)	2(8%)	5(20%)
• Fever	6(11%)	7(28%)	1(4%)
• Fever with associated symptoms	18(34%)	3(12%)	12(48%)
• URI symptoms	6(11%)	1(4%)	2(8%)
• Fatigue ,weight loss	1(2%)	3(12%)	0
• Skin rash	1(2%)	1(4%)	3(12%)
• Dizziness	1(2%)	0	0
Average duration of prodrome to neuro-symptoms	3 days	3 days	2 days
	(0–150 days)	(0–180 days)	(0–30 days)
Average duration of neuro-symptoms onset to peak	1 days	7 days	3 days
	(1–180 days)	(1–180 days)	(1–60 days)
Presenting symptoms			
• Worsening headache/neck stiffness	2(4%)	0	0
• Hemiparesis	1(2%)	0	1(4%)
• Paraparesis/paresthesia	6(12%)	3(12%)	1(4%)
• Triparesis	1(2%)	0	0
• Quadriparesis	3(6%)	1(4%)	0
• Psychomotor retardation/drowsiness	6(12%)	1(4%)	5(20%)
• Psychosis with/without seizure	2(4%)	6(24%)	0
• Behavioral change with/without seizure	13(26%)	6(24%)	12(48%)
• Seizures	19(6%)	6(24%)	4(16%)
• Facial weakness	0	0	1(4%)
• Stiff-person syndrome	0	1(4%)	0
CSF white cells range: 0–5 cells/mm^3^	26(49%)	15(60%)	5(20%)
: 6–50 cells/mm^3^	15(28%)	3(12%)	8(32%)
: 51–100 cells/mm^3^	1(2%)	3(12%)	1(4%)
: 101–500 cells/mm^3^	6(11%)	1(4%)	3(12%)
: >500 cells/mm^3^	2(4%)	0	4(16%)
CSF protein range : 0–30 mg/dl	11(21%)	7(28%)	3(12%)
: 31–60 mg/dl	19(36%)	7(28%)	6(24%)
: 61–100 mg/dl	9(17%)	5(20%)	6(24%)
: >100 mg/dl	10(19%)	3(12%)	5(20%)
CSF glucose range: 0–30 mg/dl	1(2%)	1(4%)	2(8%)
: 31–60 mg/dl	15(28%)	10(40%)	6(24%)
: 61–100 mg/dl	26(49%)	8(32%)	12(48%)
: >100 mg/dl	7(13%)	3(12%)	0
• Neuroimagings: available	44	24	23
• Normal	7(15.9%)	3(12.5%)	1(4.3%)
• Midline structures*	3(6.8%)	2(8.3%)	1(4.3%)
• Midline structures plus**	3(6.8%)	0	7(30.4%)
• White matter lesions	4(9.1%)	3(12.5%)	0
• Cortical lesions	8(18.2%)	4(16.7%)	4(17.5%)
• Multifocal lesions***	8(18.2%)	3(12.5%)	3(13%)
• Meninges	5(11.4%)	1(4.2%)	4(17.5%)
• Miscellaneous****	2(4.5%)	7(29.1%)	2(8.7%)
• Myelopathy	4(9.1%)	1(4.2%)	1(4.3%)

*midline structures referred to brainstem, thalami, basal ganglia, corpus callosum, periventricular white matter, and vermis.

**midline structures plus referred to involvement of midline structures and peripheral lesion in the cerebral hemisphere, cortical gray and white matter, subcortical white matter, and cerebellum.

***multifocal lesions referred to lesions that involved more than one of these following compartments; brain parenchyma, spinal cord and meninges.

****miscellaneous referred to non-specific lesions such as non-specific white matter changes, aging brain etc.

### Clinical profiles, course and investigation of patients with immune-mediated causes

Onset of the disease was defined as acute in 17 cases (58%), subacute 4 cases (21%) and chronic in 4 cases (21%). Common prodromal symptoms included fever alone (7, 28%) and fever with associated symptoms (3, 12%). Time between prodrome and onset of neurological symptoms varied (Table 2). Psychosis was more notable in the immune group (6, 24%). Behavioral changes were evident in all groups and seizures were found less commonly in groups lacking a confirmed etiology (only 6%).

Although fever appeared at different time points within each groups, only half of the patients (13 of 25) in the infectious group reported fever as prodromal symptom. Interestingly, fever was also found to persist despite recovery of consciousness and respiratory function in two patients in the immune-mediated group (1 anti-NMDAR and 1 anti-Yo).

CSF pleocytosis was presnt in roughly half of the patients in immune and unidentified groups and up to 80% in the infectious etiology group (Tables 2 and 3). MRI of the brain was done in all but one patient: a young male with an unstable clinical condition; CT scan of the brain was performed in this unstable patient who had anti-NMDAR antibody and the study result was normal. Abnormalities confined to temporal lobe and hippocampus suggesting limbic encephalitis were noted in one case with anti-Yo and two cases with anti-Ri antibodies.

**Table 3:** Immune-mediated encephalitis

**Clinical course & investigations**	**NMDA**	**Demyelinating diseases/NMO**	**AMPA**	**Behcet**	**ANNA2 (anti-Ri)**	**GABA**	**NMDA+ANNA2**	**VGKC**	**Anti-Yo**	**NPLE**
Encephalitis: myelitis	4: 1	1: 2	0: 1	0: 1	5: 1	1: 0	1: 0	1: 0	2: 0	3: 1
Average age (yr)	39	52	2	33	67	70	18	67	16	31
	(5–43)	(8–56)			(25–82)				(11–21)	(23–50)
Female: male	4: 1	3: 0	1: 0	2: 0	3: 3	0: 1	0: 1	0: 1	1: 1	4: 0
Underlying disease	ovarian teratoma (1)	-	-	-	DM(1), SLE(1) Ovarian cancer (1)	alcoholic cirrhosis	hemophagocytic syndrome	DM	Germ cells tumor (1)	SLE (4)
Prodrome symptoms	headache (2), fever (1)	fever (1)	fever with rash	-	fever (2), URI (1),	-	fever	fever + headache	fever (1), headache (1)	fever (2), weight loss (1), fatigue + rash + alopecia (1)
					anorexia (1)					
Presenting symptoms	psychosis + seizure (3), behavior change (1), quadripa resis (1)	behavior change ± Seizure (2), drowsy (1) parkinsonism (1)	stiff person	para paresis	psychosis ± seizure (2), Seizure (3), para paresis (1)	behavior change + Seizure (1)	seizure (1)	behavior change + Seizure (1)	behavior change (1), seizure (1)	behavior change (1), psychosis (1), seizure (1), paraparesis (1)
Average										
CSF wbc	32	5	0	157	1	0	0	0	30	0
(cells/mm^3^)	(0–62)	(5–60)			(0–19)				(0–60)	(0–1)
Average CSF protein	30	45	22	55	40	36	58	51	43	99
(mg/dl)	(19–242)	(30–66)			(2–68)				(42–45)	(28–142)
Average CSF sugar (mg/dl)	62	73	59	40	83	71	53	149	70	65
	(44–102)	(27–104)			(50–95)				(67–73)	(47–82)
Imaging pattern	normal (1), meninges (1), multifocal (2)	white matter (1), brainstem + midline (1)	normal	myelo pathy	non-specific change (2), temporal lobe (2), multiple cortical lesions (1)	white matter (1)	cerebral atrophy(1)	meninges + cortex (1)	non-specific white matter change (1), miscellenous (1)	midline + cortex (1), non-specific white matter change (1)
Outcome	complete recovered (1), partially recovered	partially recovered (1), disable (2)	partially recovered,	disable	partially recovered (4), Disable (3)	dead	partially recovered	partially recovered	partially recovered (2)	partially recovered (1), disable (2), dead (1)
	(1), disable (3)															

Table 3 summarized clinical characteristics, course of disease and results of investigations and outcome of 25 cases associated with autoimmune markers. An underlying tumor was found in 3 cases with antibodies to NMDAR (ovarian teratoma), anti-Yo (germ cell tumor) and ANNA-2 (ovarian cancer). However, another patient with anti-Yo encephalitis also had a high serum level of CA-125 despite a normal CT study of the abdomen. The clinical outcomes of immune-mediated CNS diseases varied from complete recovery in 1 case (treated with IVIG and concurrent corticosteroid), partial recovery in 10 (4 treated with immune-modulating therapy), and 12 cases who remained severely disabled (5 treated) and death in 2 cases (untreated). The following treatments were used in this series: corticosteroids were given to patients with NPLE (2), demyelinating disease (1), neuro-Behcet’s disease (1), and NMO (1); plasmapheresis was used to treat one NMDAR antibody-positive patient and IVIG with concurrent corticosteroids were used to treat 4 cases with anti-NMDAR (2) and anti-Yo (2).

Co-existence of anti-NMDAR and –Ri antibodies was found in one patient: an 18- year-old male who initially developed hemophagocytic syndrome after salmonella sepsis and was admitted to the ICU where he had seizures without focal neurological deficits. His CSF profiles were within normal limit and MRI demonstrated diffuse cerebral atrophy. The patient received intravenous antibiotics (cephalosporin) and improved over the next six weeks with partial minor disability. Result of anti-Ri and anti-NMDAR became available 3 weeks after his discharge and no immunotherapy was ever initiated. The relationship between salmonella septicemia and auto-antibodies may be coincidental. Interestingly, this case also demonstrated moderately severe cortical atrophy for his age.

Anti-Ri (ANNA2) antibody, directed against neuron oncological ventral antigens (NOVA-1 and NOVA-2) [13,14], was the most common autoantibody identified in this study (24% of immune-mediated cases). These markers were previously reported in patients presenting with opsoclonus, cerebellar ataxia, limbic encephalitis, brainstem encephalitis, myelopathy and dementia [15-17], nearly all cases in our study presented with seizures, and underlying malignancy was found in one case (ovarian cancer).

Anti-Yo (PCA-1) targeted CDR2 [18] peptides that are involved in DNA transcription. The inflammatory process against this 52 kDa antigen mediated by CD8 cytotoxic T lymphocytes [18,19] was identified in the cerebellum, brainstem, spinal cord and spinal nerve roots [20,21]. Such patients usually presented with cerebellar ataxia and associated malignancies such as ovary, breast or small cell lung cancer [15,16]. However, none of our patients presented with ataxia.

Anti-GABA_B_R-antibody has been reported in patients with limbic encephalitis (LE) associated with small-cell lung cancer (SCLC). These patients usually present with intractable seizures [22]. This antibody has been demonstrated to target the B1 subunit extracellular domain of GABA receptor [23]. This syndrome most commonly presents with subacute onset among patients in their sixth decade of life. Our case presented at 70 years of age, and with behavioral changes and seizures.

AMPAR is a subtype of glutamate receptor implicated in excitatory neurotransmission of the brain. This receptor consists of 4 subunits, but only antibodies to GluR1 and GluR2 subunits are associated with limbic encephalitis in patients with thymoma, breast cancer and lung carcinoma[24]. Most of the cases previously reported with anti-AMPAR antibody were females over 50 years of age [25], however our single patient with anti-AMPAR was a 2-year-old girl who presented with stiff-person syndrome. Anti-GAD and anti-ampiphysin antibodies in this case were negative. However, we have not tested anti-glycine receptor antibody, which has also been reported in stiff person syndrome. It is also possible that anti-AMPAR antibody in this case could be associated with viral infection suggested by the history of antecedent skin rashes.

There are 2 main targets of autoantibodies to VGKC; one at LGI1 protein and another at CASPR2 [26]. Antibodies against VGKC complexes are rarely associated with malignancies [27]. CASPR2 is largely found at the juxtaparanodal region of myelin [28]. This may explain why circulating antibody of CASPR2 can be associated with neuromyotonia, for example, as part of Morvan’s disease [29]. However, one case with anti-CASPR2 in our series manifested with solitary encephalitis instead of peripheral nervous system symptoms.

## Discussion

The results of our study confirm that autoantibody-associated encephalitis was as common as infectious encephalitis in our Thai cohort. These results are similar to the CEP result [8] and encephalitis studies from other regions [9,10,30]. However, in our study we found that this entity was not confined to young adults less than 18 years of age as previously reported [8]. The average ages of our patients with anti-NMDAR- and ANNA-2 antibodies were different; 39 and 67 years respectively. The presence of autoantibodies other than anti-NMDAR strongly support that the search for immune-mediated causes of encephalitis should not be limited to anti-NMDAR antibody alone.

Clinically, it is extremely difficult to distinguish between immune-mediated and infectious encephalitis. Immune mediated diseases such as NPLE, disease of white matter such as acute post-infectious encephalitis, NMO and neuro-Behcet’s disease should also be considered in patients with encephalomyelitis. This includes CNS vasculitis of both immune and infectious origin and mitochondrial encephalopathy, the latter of which can have MRI disturbances similar to herpes simplex encephalitis [30]. Furthermore, several autoantibodies, including those unrelated to malignancy, can be associated with this syndrome. We also could not exclude the presence of prior undetected viral, bacterial or parasitic infections that might actually have incited the aberrant immune response.

Immune-mediated encephalitis in our series was found in both sexes and in young and old patient alike (range 1-82 years of age). The majority of cases presented with an acute to subacute course. Fever was present in both immune and infectious cases of encephalitis. Psychosis was the single parameter more likely to be found in the immune mediated (24%) rather than infectious groups where there were none. Psychosis was found in 3 of 5 patients with antibody to NMDAR, 2 of 6 ANNA-2 and 1 of 4 NPLE. Seizures were found in both groups. Headache was slightly more prominent in the infectious group (20% versus 8%).

Although normocellular CSF (0-5 cells/mm^3^) can be found in 60% of immune cases (versus 20% of patients with infection), it should not be used as a solitary criteria for diagnosis because as many as 8 of 28 patients (29%) with PCR-confirmed HSV encephalitis had no CSF pleocytosis (Saraya, et al. manuscript in preparation).

Neuroimaging failed to aid in the diagnosis of immune encephalitis. Results varied and abnormalities were usually confined to the cortical and subcortical regions, whereas those associated with infection tended to involve midline and posterior fossa structures (data not shown). MRI presentation of limbic involvement, previously reported in association with antibodies against synaptic or neuronal surface proteins or intracellular antigens, was found in only 2 cases in this series.

An underlying tumor or malignancy was documented in only 3 patients. However, search for malignancy was not conducted in all cases since laboratory results were known after discharge and the patients were too disabled or the patient or families’ refused to participate in additional investigations.

## Conclusion

The results from this study should encourage physicians to aggressively screen for immune causes of encephalitis in all patients with a negative work-up for infectious encephalitis. Efforts should be made to expedite identification of autoantibodies as soon as possible, as delays in treatment may allow patients to progress to coma and/or require intensive medical care including mechanical ventilation. Ventilator associated infection or other iatrogenic complications will likely be minimized if appropriate treatment is applied in a timely manner. The high prevalence and significant morbidity of autoimmune encephalitis (24% in our study and 21% in a recent study from England) coupled to the successful treatment experience using immune-modulating therapy, make the diagnosis of immune encephalitis a priority in all patients presenting with suspected non-infectious encephalitis.

## Competing interests

The authors declare that they have no competing interests.

## Authors’ contributions

TH and SW participated in design. AS and TH participated in adult patient management. TD participated in pediatric patient management. AM and NS performed the IF laboratory assay. AS and AM participated in data collection, interpretation of the data and prepared the manuscript. TH, SS, NS, MC participated in interpretation of the data and prepared and reviewed the manuscript to final version. HW and MC contributed to and edited the manuscript. All authors read and approved the final manuscript.

## Pre-publication history

The pre-publication history for this paper can be accessed here:

http://www.biomedcentral.com/1471-2377/13/150/prepub
